# The treatment of extensive scalp lesions using coplanar and non-coplanar photon IMRT: a single institution experience

**DOI:** 10.1186/1748-717X-9-82

**Published:** 2014-03-24

**Authors:** Christian Ostheimer, Martin Janich, Patrick Hübsch, Reinhard Gerlach, Dirk Vordermark

**Affiliations:** 1 Department of Radiation Oncology, Martin Luther University Halle-Wittenberg Medical School, Dryanderstrasse 4, Halle 06110, Germany

**Keywords:** Total scalp irradiation, Intensity-modulated-radiotherapy, Angiosarcoma, Mycosis fungoides, Lymphoma of the scalp, Dosimetry

## Abstract

**Background:**

This clinical study compared four different cases of extensive scalp malignancies treated by intensity-modulated radiation therapy. The merits of coplanar and non-coplanar Step-and-shoot total scalp irradiation techniques were evaluated against the background of the literature.

**Methods:**

Four patients (angiosarcoma, n=2, cutaneous B-cell non-Hodgkin lymphoma, B-NHL, n=1, mycosis fungoides, n=1) treated between 2008 and 2012 at our institution were retrospectively analyzed. For every patient with executed coplanar plan, a non-coplanar plan and vice versa has been calculated additionally for direct comparison. Three patients underwent limited surgery before radiotherapy. Individual adapted bolus material was used for every patient (helmet). Total scalp dose was 30 Gy (B-NHL, mycosis fungoides) and 50 Gy (angiosarcoma) with fractional doses of 2.0-2.5 Gy (without sequential local boost in three patients). Conformity and homogeneity indexes and dose volume histograms were used for treatment plan comparison.

**Results:**

Dose hot spots were higher in coplanar plans (110-128% Dmax). Non-coplanar plans showed a more homogeneous dose distribution (HI = .12 - .17) and superior PTV coverage (88 - 96%). Target dose coverage was 81-117% in non-coplanar and 30-128% in coplanar plans. Coplanar plans yielded a stronger dose gradient across the target (.7-1.6 Gy/mm) compared to non-coplanar plans (.8-1.3 Gy/mm). The most conformal plan was a non-coplanar plan (CI = .7). Mean and maximum brain doses were comparable and showed an almost linear decrease between min. and max. dose. The optic chiasm and brain stem was spared most with non-coplanar plans, mean doses to the lenses ranged between 4 and 8 Gy and were higher in non-coplanar plans as were doses to the optic nerves.

Radiotherapy tolerance was acceptable and acute side effects included erythema, scalp pain, alopecia and radiodermatitis which all spontaneously resolved. Two patients accomplished partial response, two patients showed complete response after radiotherapy. Three patients had locally controlled tumors without recurrence until their deaths or at last follow up, one patient had local progression shortly after radiotherapy.

**Conclusions:**

Photon-IMRT is an effective and feasible approach to treat extensive scalp malignancies. Non-coplanar beams could increase dose homogeneity and PTV coverage and might reduce doses particularly to the optic chiasm.

## Background

Total scalp irradiation is a rather rare indication in radiation oncology, however it may be used for certain malignant lesions of the scalp including lymphoma, angiosarcoma, mycosis fungoides and squamous cell carcinoma [1-3]. Delivering an adequate target dose with a uniform dose distribution to extensive superficial scalp lesions and sparing critical structures such as the optic system and the underlying brain can be challenging due to the complex and varying geometry of the head and scalp which often is significantly altered by the presence of the tumor lesion. Traditionally, megavoltage electron beams have been used for total scalp irradiation for their finite range with a high surface dose and rapid dose falloff. Different approaches using electron fields have been described in the literature. Mellenberg et al. and Schoeppel et al. used several matching fields with performing gap shifts during treatment [4]. Able et al. achieved a more uniform dose by eliminating the gap of abutted fields and by increasing the field shift in a technique using six stationary fields [5] and Walker et al. combined tangential with normal overlapping electron fields to ensure a homogeneous dose transition between adjacent fields [6]. A similar approach utilizing overlapping fields was reported by Sagar et al. [7]. Other techniques include energy and intensity modulation of electron beams [8-10] and electron arcs, yet, with the challenge of creating an arc which entirely covers the target volume with a sufficient dose.

Apart from the complex treatment setup of the aforementioned approaches, a general limitation of electron field techniques is adequate field-matching [11].

In order to avoid these difficulties, photon beam techniques have been investigated. Akazawa et al. outlined an electron-photon technique where lateral photon fields were matched by laterally adjoining electron fields, sparing the brain with central blocks [12]. Using this method, a sufficient target volume dose could be achieved with fewer difficulties with doses at field junctions. Tung et al. took on this approach and modified it by utilizing overlapping fields instead of abutting fields, allowing a field overlap of 3–4 mm between photon and electron fields and shifting the field junction 1 cm halfway throughout the treatment course. This resulted in an improvement of dose uniformity of the technique which was initially described by Akenzawa et al. [13]. An interesting approach was reported by Kinard et al. who introduced an arcing photon shell technique, consisting of four consecutive 90-degree arcs with changing centre blocks for critical structures [14].

Intensity-modulated radiotherapy (IMRT) constitutes a promising approach in the treatment of extensive scalp lesions for its ability to homogeneously cover irregularly shaped target volumes. Lock et al. evaluated a tomotherapy-based, lateral photon-electron technique, reporting a more homogeneous dose distribution, however, at the cost of higher eye and brain doses [15]. Lower brain and eye doses with a uniform scalp dose were reported by Orton et al. who compared helical tomotherapy with a conventional lateral electron-photon technique [16]. In their treatment planning study, Bedford et al. investigated linear-accelerator-based (LINAC) IMRT in comparison with static and arcing electron techniques, using five fixed gantry angles for IMRT. Dose uniformity and target lesion coverage was superior with the IMRT approach, however, with increased but clinically acceptable doses to the eye and brain [11]. Chan et al. improved dose uniformity in the target volume while reducing doses to organs at risk such as the brain and eye by combination of photon IMRT with static electron fields compared to photon IMRT alone [17].

The necessity of a conformal bolus for adequate dose build-up outlines the challenging setup and planning requirements of IMRT-based techniques. Additionally, strict immobilization and tight treatment masks with integrated bolus limit the patient comfort in this approach. Against this background, brachytherapy is regarded as an alternative approach in the radiotherapy of scalp lesions. In the study of Imai et al., high-dose-rate (HDR) catheters were fixed to the inner layer of helmet-shaped plaster casting tape mold, placed on the head of the sitting patient [18]. In the case report of Nakamura et al., a mold made of thermoplastic material similar to the IMRT masks for head and neck cancer patients was used [19]. Despite lacking information on target volume coverage data, the two aforementioned studies reported good local tumor control and low side effects in HDR brachytherapy of angiosarcoma of the scalp. Similar results have been published by Liebman et al. for the helmet mold-based brachytherapy of chronic lymphatic leukemia [20]. In their treatment planning study, Wojcicka et al. compared dosimetric qualities of a LINAC-based photon IMRT, a helmet mold-based brachytherapy and a lateral electron-photon technique and reported superior dose homogeneity and sparing of critical structures including the optic system and the brain for the IMRT-based approach. 3D-conformal radiotherapy produced hot spots in the brain but spared optic structures. Brachytherapy in this study was accompanied by the highest doses to the brain and optic system, however, it was the most conformal treatment plan. It was concluded that HDR-brachytherapy may be an alternative treatment technique for limited scalp lesions where a lower prescribed dose is sufficient, especially for patients who are unable to receive treatment in a lying position [21].

In this retrospective study, we describe four cases of total scalp irradiation treated at our department and evaluated clinical and dosimetric outcomes against the background of the current literature.

## Methods

### Patient demographics and clinical background

**Figure 1:**
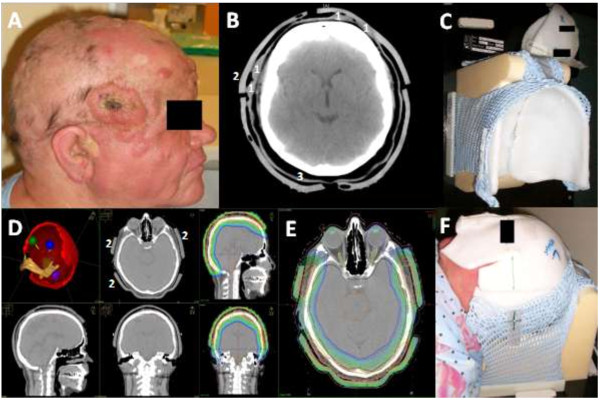
**Clinical presentation, treatment planning and setup in patient 1. (A)** Clinical presentation of patient 1 with cutaneous manifestation of T-cell lymphoma (mycosis fungoides). **(B)** Pre-treatment transversal CT scan with nodular scalp lesions (treatment planning CT with bolus in place). **(C)** Immobilization mask with integrated wax bolus. **(D)** Contured target volume, organs at risk (green/blue: eye bulb; red PTV scalp; yellow: optic nerves; purple: left parotid; orange: brain stem; beige: mandible bone), bolus and tumor lesion. **(E)** transversal cranial treatment planning CT with dose distribution over PTV from 12% isodose (red line) to 52% isodose (dark blue line). **(1)** tumor lesions, **(2)** bolus, **(3)** immobilization mask. **(F)** Complete treatment setup with correct patient positioning, wax bolus and opened immobilization mask.

**Figure 2:**
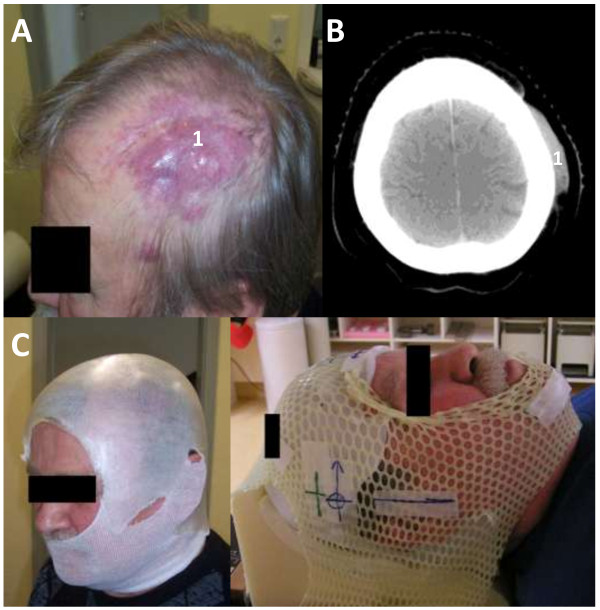
**Clinical presentation and treatment setup in patient 2. (A)** Clinical presentation of patient 2 with cutaneous manifestation of B-NHL (1) indicates tumor lesion **(B)** pre-treatment transversal CT scan with scalp tumor (treatment planning CT). **(C)** treatment setup (left) head cover and custom-made individual bolus on top (right) immobilization mask with bolus underneath.

The study is based on four patients (2 females and 2 males, median age 61 [48–89] years) with extensive scalp lesions who have been treated at the Department of Radiation Oncology, Martin Luther University Halle-Wittenberg, Germany, between 2008 and 2012. Patient, tumor and original treatment characteristics are presented in Table 1. Histological confirmation of diagnosis was obtained in all patients. Three of four patients underwent surgical resection for their tumor before radiotherapy and were treated postoperatively (adjuvant radiotherapy). One patient received radical local radiotherapy. The aforementioned patient presented with progressive cutaneous manifestation of mycosis fungoides (T-cell lymphoma) with diffuse multifocal infiltration of the entire scalp and face (Figure 1A, B) after interferon alpha and psoralen + UV-A treatment (PUVA). The second patient underwent diagnostic resection of a left-sided parietal scalp lesion and was diagnosed with cutaneous B-cell non-Hodgkin lymphoma (B-NHL). Post-surgery, two further malignant lesions remained in situ in this patient (Figure 2A, B). Among the two patients with angiosarcoma, one displayed extensive multifocal, partly exulcerated and bleeding lesions covering the entire fronto-parietal scalp which were recurrent after initial resection. The other patient was status post surgical removal of a large angiosarcoma of the left-sided vertex with osseous infiltration. Indication for radiotherapy was determined by the interdisciplinary tumor board.

**Table 1:** Patient, tumor and original treatment details

**No.**	**Gender**^**1**^	**Age (years)**	**Initial diagnosis (month-year)**	**PTV**	**Primary lesion location**	**PTV (ccm)**	**Treatment regimen**	**SD**^**2 **^**(Gy)**	**TD**^**3 **^**(Gy)**	**IMRT technique (photon 6 MV)**	**Number of couch angles not euqal 0°**	**Plan type**
1	f	53	mycosis fungoides (04–2010)	whole scalp + face	entire scalp, face	2064.7^5^	definitive RT	2	30	11 fields	0	coplanar
2	m	69	B-cell NHL^4^ (01–2009)	whole scalp	parietal left	634.5	adjuvant RT	2	30	12 fields	1 (90°)	coplanar
3	f	48	angiosarcoma (08–2009)	whole scalp	parietal left	508.8	adjuvant RT	2	50	9 fields	5 (30, 90, 330°)	non-coplanar
4	m	89	angiosarcoma (10–2010)	whole scalp	fronto-parietal	452.5	adjuvant RT	2.5	50	11 fields	6 (45, 90, 315°)	non-coplanar

^1^female (f), male (m) ^2^single dose ^3^total dose ^4^non-Hodgkin lymphoma ^5^facial tumor lesions of this patients included in PTV.

Overall, two coplanar and two non-coplanar treatment plans have been executed and for each patient with existing coplanar plan, a non-coplanar plan and for every patient with primary non-coplanar plan, a corresponding coplanar plan version has been calculated additionally for direct comparison. The newly calculated corresponding IMRT plans were based on the same PTVs, organs at risk (OAR) and prescribed doses as used in the originally carried out treatment plans.

### Treatment characteristics

All patients underwent planning computed tomography (Siemens Lightspeed RT; contrast medium was not used) and the Oncentra Masterplan External Beam planning software (Nucletron, Elekta) was used for contouring and IMRT planning.

Table 1 provides an overview of planning target volume (PTV, from skin surface to skull cap) size and location. The following organs at risk (OAR) were delineated: brain, brain stem, optic chiasm, optic structures (including optic nerves, eyes and lenses), parotid glands, mandible bone and spinal cord.

Intensity-modulated photon radiotherapy (IMRT) was carried out at the Siemens Oncor lineac-accelerator (Linac) as a “Step-and-shoot” technique with a Micro-Multi-Leaf collimator and a dose rate of 300 monitor units (MU) per minute; IMRT plans were generated with 6 MV photons. A custom-made wax bolus for the entire scalp was used for every patient to ensure sufficient surface dose for IMRT treatment and was integrated into the individually designed thermoplastic immobilization mask in order to achieve a reproducible position of the patient, Figures 1 B-F and 2C.

Treatment planning CT studies were performed with the bolus and immobilization mask in place, Figure 1B. The maximum air gap between bolus and scalp surface for each individual IMRT treatment plan has been calculated and is reported in mm. Mean treatment duration per session is described in minutes and the dose gradient across the target volume is reported in Gy per mm. Portal imaging was utilized for correct positioning and the patients’ position was well reproduced. All four patients received a normofractionated (5 Fx/week) Linac-based IMRT which was delivered to the entire scalp, using multiple individually collimated photon standing fields. Individualized beam angles for IMRT were chosen to maximally avoid OARs. Characteristics of originally executed treatment plans are given in Table 1.

Patient 1 was given 30 Gy in 15 fractions with 11 intensity-modulated photon standing fields (72 segments, 1651 MU, 826 MU/Gy, 11 beam angles) and the corresponding non-coplanar plan was calculated utilizing 11 beam angles (59 segments, 1042 MU, 521 MU/Gy) with 6 couch angles not equal to 0° (45°, 315°, 90°). Treatment for patient 2 consisted of a regular 12-field-photon-IMRT (2–30 Gy, 84 segments, 1036 MU, 518 MU/Gy, 12 beam angles) to the entire scalp (corresponding non-coplanar plan: 11 beam angles, 71 segments, 1089 MU, 544.5 MU/Gy, 6 couch angles ≠ 0°: 90°, 315°, 45°). Patient 3 was treated with a photon-IMRT (9 standing fields, 94 segments, 987 MU, 494 MU/Gy, 9 beam angles) and a fraction size of 2 Gy, given in 25 fractions to the entire scalp. A corresponding coplanar treatment plan was calculated for patient 3 using 11 beam angles, 69 segments, 1016 MU, 508 MU/Gy and all couch angles equal to 0°. 50 Gy in 20 fractions were given to the whole scalp of the fourth patient, using 11 photon-IMRT standing fields (86 segments, 1143 MU, 457 Mu/Gy, 11 beam angles). For the same patient, a corresponding coplanar plan was calculated using 11 fields (68 segments, 1069 MU, 428 MU/Gy) with all couch angles equal to 0°. Three of the four patients received a sequential locally circumscribed tumor boost with 5 × 2 Gy (patient 2 and 3) and 8 × 2.5 Gy (patient 4) using two 9 MeV electron fields (patient 2 and 4) and a 3D-conventional radiation technique (with bolus, 6 MV, 3 fields, patient 3), respectively.

### Dose conformity

An overview of different conformity indexes has been published by Feuvret et al. [22].

In our study, we used the conformity index (CI) as defined by Paddick et al. [23]:

(1) $$\mathrm{CI}=\frac{\mathrm{TV}{\left(\mathrm{PIV}\right)}^{2}}{\mathrm{TV}\times \mathrm{PIV}}$$

where TV was the target volume and PIV the prescription isodose volume, referring to the 95% or 98% isodose. TV(PIV) describes the target volume covered by the prescription isodose volume. The homogeneity index (HI) was defined in accordance with the ICRU report 83 [24-26] as

(2) $$\mathrm{HI}=\frac{D_{2}-D_{98}}{D_{50}}$$

with D_2_ and D_98_ representing the doses received by 2% and 98% of the PTV and D_50_ being the median absorbed dose. PTV coverage was the ratio of the PTV receiving 95% of the prescribed dose (V_95%_) and the corresponding PTV (23):

(3) $$\mathrm{PTV}\;\text{coverage}=\frac{\mathrm{TV}\left(95\%\right)}{\mathrm{TV}}$$

## Results

### Dosimetric analysis of IMRT treatment plans

Dosimetric comparison of the four IMRT plans and their corresponding newly calculated plans including PTV coverage, homogeneity, conformity and OAR involvement is presented in Table 2 and refers to the primary radiotherapy given to the patients (without tumor boost) as described in Table 1.

**Table 2:** Planning target volume homogeneity, conformity, dosimetry and organ at risk doses in non-coplanar versus coplanar IMRT plans

**No.**^**1**^	**Planarity**^**2**^	**HI**^**3**^	**CI [95%]**^**4**^	**CI [98%]**^**4**^	**PTV coverage**^**5**^	**PTV Dmax**^**6 **^**Gy (%)**	**PTV Dmin Gy (%)**	**Max. air gap mm**	**Bolus size mm**	**Treatment duration**^**7 **^**mean min**	**Dose gradient Gy/mm**	**Mean brain dose Gy (%)**	**Max. brain stem dose Gy (%)**	**Max. optic chiasm dose Gy (%)**	**Max. optic nerve dose**^**8 **^**Gy (%)**	**Mean lens dose**^**8 **^**Gy (%)**	**Max brain dose Gy (%)**
1	coplanar	.25	.66	.54	.83	38.3	9	8	8	15	1.05	13.3	23.5	11.2	14.3 (47.8),	4.7 (15.6),	30.4
						(127.7)	(30)					(44.3)	(78.3)	(37.4)	11.3 (37.7)	5.9 (19.7)	(101.3)
	*non-coplanar*	.17	.69	.65	.88	34.8	20.8	8	8	n/a	.92	15.2	20.5	5.3	9 (30),	7.6 (25.3),	31.1
						(115.9)	(82.7)					(50.8)	(68.2)	(17.7)	8.3 (27.7)	8.4 (28)	(103.6)
2	coplanar	.21	.45	.42	.84	34.9	23.7	8	7	16	.7	14.4	16.5	7.5	10.6 (35.2),	4 (13.2),	31.7
						(116.4)	(79)					(47.9)	(55.1)	(24.9)	10.9 (36.3)	4.1 (11.9)	(105.7)
	*non-coplanar*	.15	.56	.47	.88	33.8	24.4	8	7	n/a	.8	14.5	12.5	3.2	13.4 (44.7),	4.8 (16.1),	32.2
						(112.8)	(81.3)					(48.2)	(41.6)	(10.7)	11.4 (38)	5.2 (17.3)	(107.4)
3	non-coplanar	.17	.47	.42	.88	56.9	40.4	5	8	18	1.25	21.4	17.5	7.7	20.8 (41.6),	7.5 (14.9 ),	53.5
						(113.8)	(80.8)					(42.8)	(35.1)	(15.5)	21.8 (43.6)	7.8 (15.6)	(107)
	*coplanar*	.18	.54	.45	.86	57.8	41.5	5	8	n/a	1.59	23.2	15.8	14.2	18.3 (36.6),	5.8 (11.6),	53.6
						(115.6)	(83)					(46.3)	(31.6)	(28.4)	17.6 (35.1)	5.9 (11.7)	(107.2)
4	non-coplanar	.12	.49	.45	.96	58.5	42.3	3	5	19	1.17	23	3.7	4.4	24.8 (49.5),	6.7 (10.3),	52.5
						(116.9)	(84.6)					(46)	(7.4)	(8.8)	23.5 (46.9)	5.9 (11.9)	(105)
	*coplanar*	.14	.59	.51	.92	55.1	40	3	5	n/a	1.42	24.8	10	9.1	20.5 (41),	6.5 (12.9),	54.5
						(110.3)	(80)					(49.7)	(20)	(18.2)	19.6 (39.3)	7.6 (15.2)	(109.1)

^1^patient number ^2^the upper line refers to the originally executed treatment plan and the lower line to the additionally calculated corresponding plan using opposite planarity for direct comparison ^3^homogeneity index according to ICRU 83 ^4^conformity index according to Paddick et al. referring to 95% and 98% isodose ^5^ratio of V95 and corresponding PTV ^6^dose hot spot ^7^per session in originally carried out treatmemt plans ^8^right, left.

Originally, a non-coplanar plan was executed for patient 3 and 4; patient 1 and 2 received a coplanar technique.

Mean prescribed doses (referring to the 107% isodose) were 30 Gy for patient 1 (mycosis fungoides) and patient 2 (B-cell NHL) and 50 Gy for patient 3 and 4 (angiosarcoma), Table 1.

Maximum PTV doses of originally executed plans were 38.3 Gy (patient 1), 34.9 Gy (patient 2), 56.9 Gy (patient 3) and 58.5 Gy (patient 4). All four plans produced dose hot spots over 100% which were higher in the coplanar plans (110 - 128% of PTV dose) except for one patient, Table 2. Mean daily treatment duration was longer for non-coplanar techniques (18 and 19 minutes) as opposed to the coplanar plans (16 and 15 minutes) because the couch angle had to be changed manually, Table 2. Maximum air gaps between bolus and target volume were 5 and 3 mm in the non-coplanar plans and 8 mm in the coplanar plans. In the originally executed plans, coplanar techniques yielded a stronger dose gradient across the target (1.1, 1.6 and 1.4 Gy/mm) than the coplanar techniques (.9, 1.3 and 1.2 Gy/mm) except for one patient (coplanar .7 Gy/mm vs. non-coplanar .8 Gy/mm), Table 2.

In all four cases, the optic system (including optic nerves and chiasm) received a mean dose less than 25 Gy. In all patients, non-coplanar plans provided lower doses to the optic chiasm (18%, 11%, 16% and 9% of the mean PTV dose) compared to the coplanar plans (37%, 25%, 28% and 18%, Table 2) but optic nerves were spared most in the coplanar plans except for patient 1 whose left and right optic nerve received less dose in the non-coplanar plan version (30% and 28% vs. 48% and 38%, right and left optic nerve, Table 2). Mean doses to the lenses ranged between 4 and 8 Gy which was not exceeded and were higher in the non-coplanar plans (10 - 28% of PTV dose) compared to coplanar plans (12 - 20%), except for patient 4 (non-coplanar 10% and 12% vs. coplanar 13% and 15%, Table 2). Maximum brain stem doses were higher in the coplanar treatment plans (78%, 55%, 20%) as opposed to the non-coplanar plans (68%, 42%, 7%) except for patient 3 whose coplanar plan provided a lower brain stem dose (32% vs. 35%), Table 2.

**Figure 3:**
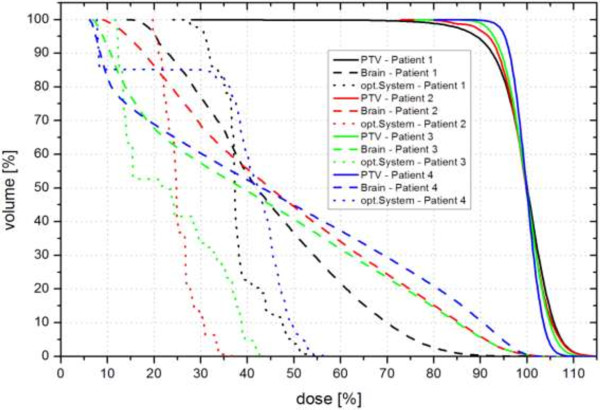
**Comparative dose-volume-histogram (DVH) for patient 1-4.** DVH of patient number 1 (black), 2 (red), 3 (green) and 4 (blue) with dose-volume relation of planning target volume (PTV, continuous lines) and organs at risk brain (dashed lines) and optic system (incl. optic nerves and chiasm, dotted lines); each line represents one patient; axis of abscissae with dose (%), ordinate with volume (%).

Mean doses to the brain did not go beyond 25 Gy and were comparable between coplanar and non-coplanar plans: in two patients, non-coplanar plans produced lower mean brain doses (43% vs. 46% vs. and 46% vs. 50%), in the one patient, the coplanar plan spared the brain better (44% vs. 51%) and in another patient, mean brain doses were almost identical, Table 2. Maximum brain doses based on mean PTV dose in originally executed plans were 30 Gy (101%, patient 1), 32 Gy (106%, patient 2), 54 Gy (107%, patient 3) and 52 Gy (105%, patient 4) and showed an almost linear dose decrease between min. and max. dose which is shown in a comparative dose volume histogram (DVH), Figure 3. Direct comparison between the originally executed plans and their newly calculated corresponding versions showed higher maximum brain doses in the non-coplanar plans of two patients (104% and 107%) compared to their corresponding coplanar versions (101% and 106%). In one patient, maximum brain doses were almost identical in both plan versions (patient 3) and in another patient, the non-coplanar plan spared the brain better than its coplanar counterpart (105% vs. 109%, patient 4), Table 2.

The dose-volume-relationship of PTV and the OAR brain and optic system of patient 1 to 4 is illustrated in a comparative DVH, Figure 3.

In all patients, non-coplanar plans yielded a more homogeneous dose distribution (HI = .17, .15, .12) compared to the coplanar plans (HI = .25, .21, .18, .14) and dose distribution was 81-117% for non-coplanar plans while it was 30-128% in the coplanar plans. PTV coverage was superior in the non-coplanar plans (88% and 96%) when compared to coplanar plans (83%, 84%, 86% and 92%), Table 2.

In two patients, the non-coplanar plans were more conformal than their coplanar counterparts (CI = .69 and .56 vs. CI = .66 and .45) while in the other two patients, conformity was higher in the coplanar plans (CI = .54 and .59 vs. CI = .47 and 49), Table 2.

### Clinical outcomes of patients

There were no complications identified during treatment and visible tumor regression was already noted intratherapeutically in all four patients.

Acute side effects included radiodermatitis (CTC grade II-III) and erythema (CTC grade I-II) of the skin which resolved within one month after the end of radiotherapy. Grade I scalp pain and alopecia (CTC grade II) was observed, however these conditions resolved within 3 months after radiotherapy. Good response to radiotherapy with a marked regression of malignant skin lesions was noted in all patients after radiotherapy at the first post-treatment follow-up evaluation.

**Figure 4:**
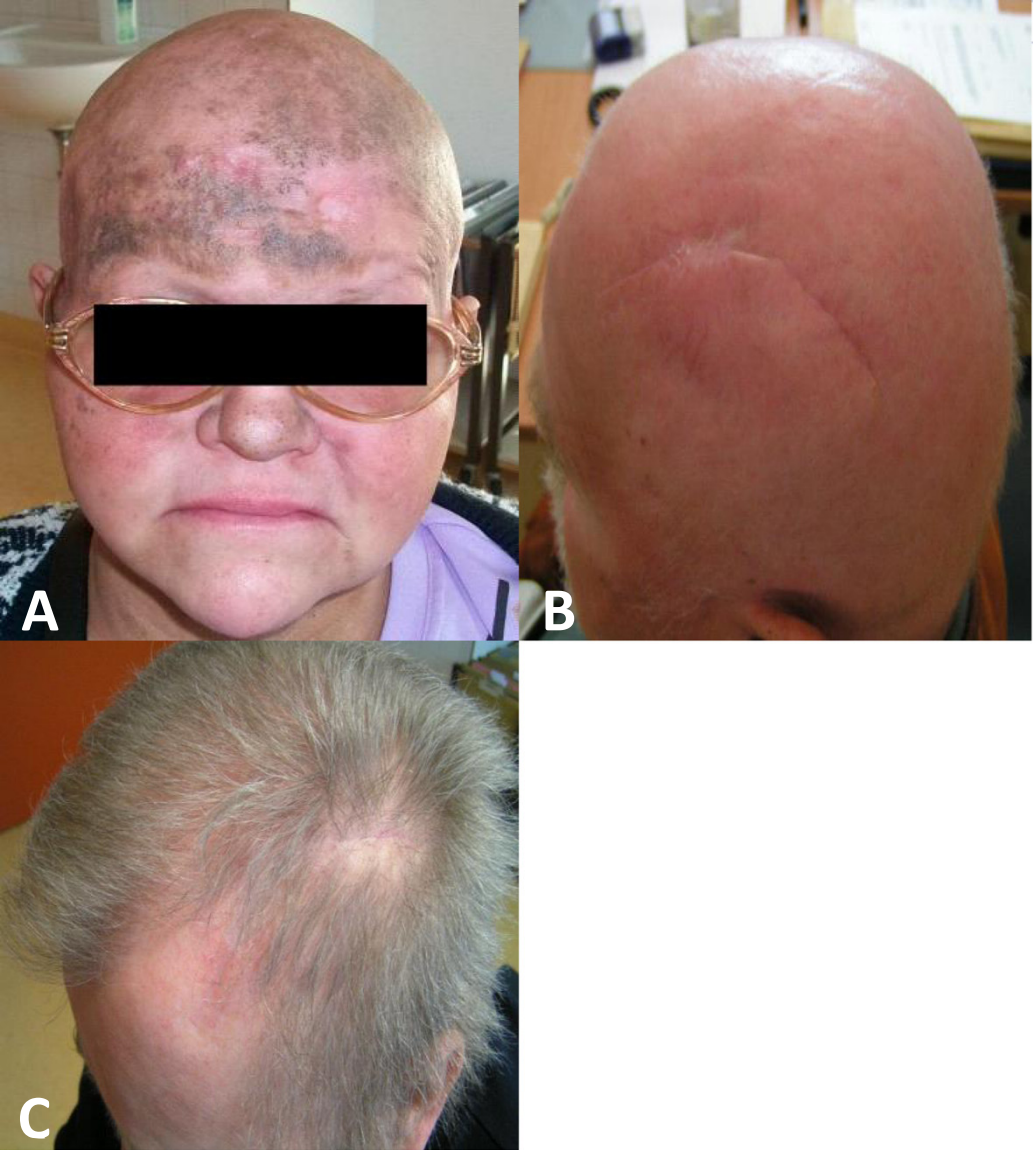
**Clinical outcome after completion of radiotherapy.** Post-treatment evaluation four weeks after the end of radiotherapy in **(A)** patient 1 and **(B)** patient 2. Clinical presentation one year after radiotherapy in patient 2 **(C)**.

Patient one developed edema, erythema and puffiness of the irradiated skin but initially (at the first post-radiotherapy follow-up) showed good treatment response with at least partial remission of the visible mycosis fungoides lesions, Figure 4A. Unfortunately, the same patient suffered from general disease progression with whole body involvement 5 months after completion of radiotherapy which resulted in the death of the patient. After the end of radiotherapy, patient 2 had achieved complete remission and a low-grade erythema of the scalp with alopecia CTC grade II was noted but resolved within 1 month after treatment with minor telangiectasis remaining in the irradiated skin area, Figure 4B. One year after radiotherapy, hair growth was grossly restored (Figure 4C) and at the most recent follow-up 4 years after radiotherapy, the patient was clinically stable without any evidence of recurrent disease. Patient 3 also showed complete macroscopic remission four weeks after the end of radiotherapy and the tumor remained controlled without evidence of recurrent disease until the death of the patient 27 months after radiotherapy. In patient 4, partial remission with clear regression of the pre-therapeutically visible tumor lesions was noted after the end of radiotherapy. Unfortunately, the same patient presented with a massive local disease progression one month after completion of radiotherapy.

## Discussion

Extensive malignant lesions of the scalp constitute a rare indication in radiation oncology but owing to their complex and varying presentation, they often are associated with considerable treatment planning and setup efforts in order to deliver an adequate and homogeneous dose to the target volume while sparing critical structures. Low prevalence and small sample size additionally limit large-scale investigations and complicate the evaluation and selection of appropriate radiotherapy approaches. Since complex field matching (by junction shifting for instance) is required for electron-based techniques, photon-IMRT is considered a good alternative in the total radiation of the scalp besides photon-electron combinations and HDR brachytherapy in certain cases.

In this work, a series of four patients with extensive scalp lesions treated with photon-IMRT using different planarity is reported and evaluated.

Non-coplanar IMRT plans delivered better results of PTV coverage and dose homogeneity compared to coplanar plans where dose distribution was less homogeneous (dose gradient in the target 30-128% compared to 81-117% in non-coplanar plans). The non-coplanar plan of patient 1 (mycosis fungoides) was the most conformal plan (CI = .7); in two patients, non-coplanar plans were of superior conformity while in the other two patients, coplanar plans were more conformal, however, with the constraint of the limited informative value of the CI [23].

Doses at critical organs, particularly doses to the brain and the optic system were clinically acceptable and are comparable with current literature [11,15]. Woicicka et al. reported slightly improved DVHs of the optic structures with non-coplanar beams [21]. In our study, coplanar plans produced higher OAR doses for the optic chiasm and brain stem but doses to the lenses and the optic nerves were higher in non-coplanar plans (except for one patient). IMRT is reported to only slightly increase doses to optic structures [11,21] which might be reduced by the use of non-coplanar beams, especially for the optic chiasm as our results suggest.

In general, mean and maximum brain dose was comparable for coplanar and non-coplanar techniques: maximum brain dose of the coplanar and non-coplanar plan was almost identical in one patient whereas in two patients, it was higher in the non-coplanar plans and in one patient, the non-coplanar plan provided a lower maximum brain dose. In two patients mean brain dose was higher in the coplanar plans, in the remaining two patients, non-coplanar plans yielded a higher mean dose to the brain.

Except for one patient, dose hot spots were lower in non-coplanar plans and the highest dose maximum was produced by a coplanar plan (Dmax = 128%) while the dose gradient was superior in coplanar plans.

In the literature, helical tomotherapy also has been suggested as a promising means of delivering total scalp irradiation [15,16]. The clear advantage of this technique can be seen firstly, in its ability to deliver radiation in a tangential manner to every point of the scalp and secondly, in the avoidance of complex field matching [27]. Another potential benefit of tomotherapy is that there is no need to use different modalities such as photons/electrons [16,28]. However, there are works which report an overestimation of the superficial dose with the tomotherapy planning system [29] and some studies could not confirm the superiority of helical tomotherapy over other current approaches such as lateral photon-electron technique [15]. Locke et al. for instance compared the aforementioned technique with tomotherapy-IMRT in a patient with Merkel cell carcinoma and found that critical structure doses were unacceptable with tomotherapy although the IMRT plan provided better dose homogeneity and target volume coverage [15]. However, in the work of Locke et al., only partial and not total scalp irradiation was evaluated and several other studies support the advantageous role of tomotherapy in total scalp irradiation (i.e. improved target dose homogeneity and critical structure dose) which is also attributed to the use megavoltage computed tomography for simultaneous setup verification [16,29].

In a clinical perspective, post-treatment outcome evaluation in our study showed good response rates with successful reduction of local tumor burden in all four patients. Three of four patients had a locally controlled tumor until their deaths, one patient suffered from early local disease progression after radiotherapy.

## Conclusion

In summa, our data underlines that photon-IMRT offers an effective and feasible treatment approach for extensive and complex shaped scalp lesions [1] with acceptable OAR doses, controllable side effects and satisfying cosmetic results. Non-coplanar IMRT plans produce lower dose hot spots and offer better dose homogeneity, better target volume coverage and lower doses to the optic chiasm and brain stem than coplanar plans, however, at the cost of longer treatment times, higher doses to the optic nerves and lenses, complex setup and setup uncertainty, which is increased by the sharp dose gradient [11,30].

## Consent and ethical approval

A positive vote for this study was given by the ethics committee of the Medical Faculty of the Martin Luther University Halle-Wittenberg and all research has been carried out in compliance with the Helsinki Declaration. Written informed consent was obtained from all patients for the publication of this report and any accompanying images.

## Competing interests

On behalf of all authors, the corresponding author declares that there are no competing interests.

## Authors’ contributions

CO and DV conceived of the study. RG and DV participated in its design and coordination. CO carried out data acquisition, participated in the sequence alignment and drafted the manuscript. MJ and PH carried out treatment plan and dosimetric calculation. All authors read and approved the final manuscript.
